# Strategies to improve healthcare services for patients with sickle cell disease in Nigeria: The perspectives of stakeholders

**DOI:** 10.3389/fgene.2023.1052444

**Published:** 2023-02-03

**Authors:** Hezekiah Isa, Emmanuel Okocha, Samuel Ademola Adegoke, Uche Nnebe-Agumadu, Aisha Kuliya-Gwarzo, Alayo Sopekan, Akinyemi Olugbenga Ofakunrin, Ngozi Ugwu, Abdul-Aziz Hassan, Chinatu Ohiaeri, Anazoeze Madu, Ijeoma Diaku-Akinwumi, Lilian Ekwem, Livingstone Gayus Dogara, Dorothy Okoh, James Jasini, Ahmed Girei, Timothy Ekwere, Angela Okolo, Umar Kangiwa, Juliet Lawson, Reuben Chianumba, Biobele Brown, Norah Akinola, Maxwell Nwegbu, Obiageli Nnodu

**Affiliations:** ^1^ Centre of Excellence for Sickle Cell Research and Training, University of Abuja (CESRTA) Federal Capital Territory (FCT), Abuja, Nigeria; ^2^ Department of Haematology and Blood Transfusion, University of Abuja, Abuja, Nigeria; ^3^ Department of Haematology, Nnamdi Azikiwe University Teaching Hospital Nnewi, Nnewi, Anambara State, Nigeria; ^4^ Department of Paediatrics and Child health, Obafemi Awolowo University, Ile Ife, Osun State, Nigeria; ^5^ Department of Paediatrics, University of Abuja Teaching Hospital, Abuja, Nigeria; ^6^ Department of Haematology, Aminu Kano University Teaching Hospital, Kano, Kano State, Nigeria; ^7^ Non Communicable Disease Unit, Federal Ministry of Health, Federal Secretariat, Abuja, Nigeria; ^8^ Department of Paediatrics, Jos University Teaching Hospital, Jos, Nigeria; ^9^ Department of Haematology, Alex Ekweme Federal Teaching Hospital, Abakaliki, Ebonyi State, Nigeria; ^10^ Department of Haematology and Blood Transfusion, Ahmadu Bello University, Zaria, Kaduna State, Nigeria; ^11^ Department of Paediatrics, Federal Medical Centre Keffi, Keffi, Nasarawa State, Nigeria; ^12^ Department of Haematology, University of Nigeria Teaching Hospital, Enugu, Enugu State, Nigeria; ^13^ Department of Paediatrics, Lagos State University Teaching Hospital, Ikeja, Lagos State, Nigeria; ^14^ Department of Paediatrics, General Hospital Nyanya, Abuja, Nigeria; ^15^ Departments of Haematology and Blood Transfusion, Kaduna State University Teaching Hospital, Kaduna, Kaduna State, Nigeria; ^16^ Department of Haematology, Rivers State University, Port Harcourt, Rivers State, Nigeria; ^17^ Department of Haematology, Federal Medical Centre, Yola, Adamawa State, Nigeria; ^18^ Department of Haematology, Federal Teaching Hospital Gombe, Gombe, Gombe State, Nigeria; ^19^ Department of Haematology, University of Uyo, Uyo, Akwa IbomState, Nigeria; ^20^ Federal Medical Centre Asaba, Asaba, Delta State, Nigeria; ^21^ Department of Haematology, Federal Medical Centre, Birnin Kebbi, Kebbi State, Nigeria; ^22^ Department of Paediatrics, Zankli Medical Centre, Abuja, Nigeria; ^23^ Department of Paediatrics, University College Hospital, Ibadan, Oyo State, Nigeria; ^24^ Department of Haematology, Obafemi Awolowo University Teaching Hospital, Ile Ife, Osun State, Nigeria; ^25^ Department of Chemical Pathology, University of Abuja, Abuja, Nigeria

**Keywords:** healthcare services, sickle cell disease, stakeholder engagement, Nigeria, sickle cell disease network

## Abstract

**Background:** Sickle cell disease (SCD) continues to pose physical and psychosocial burdens to patients, caregivers and health workers. Stakeholder engagement in the processes of policy making and implementation is increasingly becoming the cornerstone of best practices in healthcare.

**Aim and Objectives:** To engage stakeholders with a view to assessing the knowledge of SCD; ascertain the challenges associated with accessibility and affordability of healthcare services; improve the quality of care, and thereby effect behavioral change through increasing attendance and follow-up of patients in the clinics.

**Methodology:** A Stakeholders’ Engagement meeting organized by the Sickle Pan Africa Research Consortium Nigeria Network (SPARC-NEt) was attended by patients, caregivers and members of patient support groups, healthcare providers and management/policymakers. The engagement was through PowerPoint presentations, structured questionnaires and an interactive session. The structured questionnaire assessed the knowledge of stakeholders about SCD; the quality of healthcare services; challenges with access and affordability; and SCD-related government policies.

**Results:** Three hundred and twelve stakeholders attended the engagement meeting. Of the 133 that participated in the study, medical workers were the most represented. The majority had good knowledge of what causes SCD (96.2%) and the best place to get help during SCD crisis (98.5%). However, knowledge of the specific preventive measures of SCD and its crisis was not optimal. In terms of the role of community engagement and education, only about one-quarter of the study participants, 34 (25.6%) knew about their positive role in reducing the prevalence of SCD and alleviating SCD crises. Challenges identified include inadequate healthcare personnel and facilities, delay in obtaining laboratory results, long waiting time in the clinic, poor communication, absence of holistic consultation, uncoordinated healthcare services, high cost of care, ignorance, non-prioritization of SCD by government, lack of multisectoral collaboration and partnership with NGOs and international organizations. Strategies proffered to improve healthcare services include, community/stakeholder engagement and health education, sickle cell daycare services, access to a willing and dedicated multidisciplinary workforce, collaboration with support groups and government policies and programs.

**Conclusion:** There is need for regular stakeholder engagement to improve access to healthcare services for SCD patients in Nigeria.

## Introduction

Sickle cell disease (SCD) is highly prevalent in Nigeria. According to the National Demographic Survey 2018, 20% of children aged 6–59 months have sickle cell trait [SCT (HbAS)] and 1.5% have haemoglobin C trait (HbAC), while the prevalence of some types of SCD (HbSS and HbSC) among children is 1.2% (National Population Commission (Nigeria) and ICF, 2019). SCD is a life-long disorder associated with morbidity and mortality (Isa et al., 2020). The curative therapy is bone marrow transplantation which is available in the country but is largely unaffordable by the majority of patients (Adewoyin, 2015). Few simple evidence-based interventions have been shown to reduce the morbidity and mortality of SCD in developed countries. These, along with education of the patients and caregivers on how to maintain good health has been impactful in low-income settings (Bello-Manga et al., 2020).

The government on its part has taken some measures towards providing quality healthcare services for SCD such as the development of the National Guideline for the Management and Control of SCD (The federal ministry of health, 2014), establishment of six zonal Centres of Excellence for SCD equipped with HPLC for early detection and comprehensive care, creation of a National Desk for SCD at the Federal Ministry of Health (FMoH) and more. Clinical care is available for patients with SCD mainly in the secondary and tertiary healthcare facilities but the majority of patients with SCD remain unreached in the rural communities of Nigeria. (Nnodu, 2014)^,^ (Adegoke et al., 2018) Access to healthcare, client expectations from services, practices of healthcare providers and the government or institutional policies require appraisal, hence the need to engage stakeholders**. S**takeholder engagement has been used to assist with the processes of policy making and implementation is increasingly becoming the cornerstone of best practices in healthcare (Culyer, 2005; Wu et al., 2019).

The Sickle Pan Africa Research Consortium NigEria Network (SPARC-NEt) comprises 25 healthcare centers across the six geopolitical zones of Nigeria with the hub at the Centre of Excellence for Sickle Cell Research and Training, University of Abuja (CESRTA). SPARC-NEt is a member of a larger consortium, the Sickle Pan African Research Consortium (SPARCo) comprising six sub-Saharan African countries: Tanzania, Ghana, Nigeria, Mali, Uganda, Zambia/Zimbabwe. The main objectives of SPARCo are to expand the existing sickle cell registry which currently has over 13,000 patients; establish and implement a uniform multi-level standard of healthcare for sub-Saharan Africa; strengthen existing skills, and undertake collaborative research in SCD in a sustainable manner.

The SCD stakeholders’ workshops were organized to engage critical stakeholders (patients, caregivers, health workers and policymakers) on the nature of SCD, ascertain the challenges that the patients face in accessing care, find the best way to provide services, increase the number of patients who are enrolled and followed up actively in the clinics at the SPARC-NEt sites. For the purpose of this study, stakeholders refer to entities that are integrally involved in the healthcare system and are substantially affected by reforms to the system. At the end of the workshop, participants were expected to know more about SCD and the importance of regular health maintenance which could lead to behavioral change with consequent improvements in patient outcomes.

## Methodology

SPARC-NEt organized a hybrid (physical and virtual) stakeholder engagement workshop on the 24th of February 2022. Each site was expected to host about 18 participants comprising 10 patients/caregivers and eight other stakeholders including doctors, nurses, pharmacists, medical laboratory scientists, medical and health records staff, research assistants, other patient support groups members, management staff/policymakers and the media. Participants met in person (physically) at each site from where they joined the virtual meeting hosted at the SPARC-NEt hub at the University of Abuja *via* a ZOOM link provided to the Site Leads. Participants who could not join other stakeholders physically at the various sites joined remotely from wherever they were using the ZOOM link.

Stakeholders were engaged through PowerPoint presentations and survey questionnaires which was followed by an interactive session. The presentations from patients/caregivers and other patient support group members focused on challenges in accessing healthcare services and their expectations from other stakeholders. Healthcare providers (doctors, nurses, pharmacists, health record staff, and laboratory staff) focused on practice experiences and role of education in the management of SCD, while Management/policymakers focused on policies and government plans for SCD in Nigeria in their various presentations. For the survey, a structured questionnaire was administered electronically and hard copies were made available for stakeholders who had no access to the electronic version. The questionnaire had structured questions that assessed the knowledge of the stakeholders about SCD. Ethics approval, (NHREC/01/01/2007–24/11/2021D) was obtained from the National Health Research Ethics Committee (NHREC) of Nigeria.

Participation was voluntary, consent was inferred for those who completed the questionnaire and the data collected from the questionnaires were anonymous. Stakeholders who made presentations consented to use their presentations for purpose of research and publication. The presentations were summarized in tabular form and data from the questionnaire was presented using descriptive analysis on IBM SPSS software version 23.

## Result

A Perspective of stakeholders
B Result of survey

## Summary of results

Table 1 shows the composition of the stakeholders who attended the workshop. Close to 50% were patients/caregivers, healthcare providers constituted 43.4%, management/policymakers made up 5.1% while patient support groups, the media and other stakeholders were about 10%

**TABLE 1:** Workshop attendance.

Stakeholder	Number in attendance (%)
Patients/Caregivers	143 (45.8)
Doctors	65 (20.8)
Nurses	29 (9.3)
Pharmacists	10 (3.2)
Data clerks	2 (0.6)
Medical Laboratory Scientists	13 (4.2)
Health Records staff	6 (1.9)
Management/Policy makers	16 (5.1)
Research assistants	3 (1.0)
Patient support group members	8 (2.6)
Media	6 (1.9)
Project staff	5 (1.6)
Others^*^	6 (1.9)
Total	312 (100)

*Others refer to data clerks, research assistants.

Table 2 highlights the detailed feedback from patients, caregivers and patient support NGOs. The Table shows the various challenges and expected solutions in accessing healthcare, high cost of care, delay in communication with healthcare providers, absence of holistic consultation and general problem with healthcare service coordination.

**TABLE 2:** Quality of healthcare services (Feed-back from Patients/Care Givers/Patients) Support NGOs).

Experience/Challenges	Strategies/Expectations
**Access**	• Provision of more specialized sickle cell centres
• Delay before accessing care. Quantified to be about 2 h or more before talking to a doctor. Turnaround time for investigations can take up to 3 days for one consultation	
• Difficulty in accessing a preferred healthcare worker	• Capacity building, enhancement, and job protection for health workers to enable them put in their best
• Healthcare workers are unfriendly	
• Specialist SCD centres are few and difficult to access	
• Unorthodox, and uncertified care is very much available and easy to access	
**Contextual knowledge (knowledge of healthcare providers on needs of patients)**	• Every clinic day should be a unique journey with exciting new things to look forward to
• Family support is poor	
• Clinic days are too routine and rigid and often difficult to fit into individual patient’s schedules	
**Communication**	• Provision of more specialized sickle cell centres and employment of more healthcare providers especially social workers and haemoglobinopathy counsellors
• Healthcare workers are too much in a hurry to write drugs and dismiss a patient without listening to the problems of the patient	
• The guinea pig mentality. Sometimes patients believe that everything done for the patient is just to make him/her give blood for research	
**Comprehensiveness**	• Multidisciplinary team management
• Consultations not holistic as social and spiritual issues are seldom attended to	
• Lack of counselling that may lead to the philosophy “once l am fine, no need to go to the clinic”, self-medication and patronage of quacks may then follow	• Health education and public enlightenment for all
• Stigma	
**Coordination**	• Improvement of emergency care and blood transfusion services
• Emergency care and blood transfusion are inefficient	
• The National Health Insurance Service (NHIS) is unfriendly to patients. Many times, routine drugs for SCD are not available in the scheme or out of stock	• Government should make NHIS more friendly and effectively accommodate sickle cell patient
**High cost of services**	• Increase health insurance coverage and inclusion of hydroxyurea in NHIS drugs list
• Cost implications, an average of 10,000 naira ($20) on drugs monthly, excluding transport, investigations, consultation fee and others	
• Important drugs for care such as hydroxyurea are expensive and difficult to access	

Table 3 shows the feedback from healthcare providers. It highlights best practices for SCD care including roles of community/stakeholder engagement and health education, sickle cell day care services, access to a willing, dedicated and trained multidisciplinary workforce, roles of SCD registry, and collaboration with support groups that provide basic materials for care such as fluids, analgesics, antimalarial, and others.

**TABLE 3:** Feedback from healthcare providers (doctors, nurses, pharmacist, medical laboratory scientists, and health records staff).

Practice experience/Best practices
**Health Education**
• Health education (HE) is a very important component of care. Promotes wellbeing through behavioural change
• HE begins at diagnosis and is continued at every clinic visit
• Has various components: Education about the disease, health maintenance strategies, psychosocial issues, and genetic counselling
• Emphasize component at appropriate age
• Should be evaluated
Practical outcomes
Beneficial increase in family knowledge of SCD and health literacy levels, ability to resist prodding for unorthodox practices, participation in self-care, better health seeking behavior and overall improvement in quality of life and survival
**The Sickle Cell Day Care Service Model (Day Care Centre)**
• Strategically located within the hospital
• Provides short term emergency care which cuts down length of stay and cost
• Provides easy access to services
• Has management representation and a multidisciplinary team
• Ease of co-ordination
• Support skills services (counsellors, record clerks, side laboratory, pharmacy and others)
**Other effective evidence-based practices**
• Access to a willing, dedicated and trained multidisciplinary workforce
• Know your patient - profiling of patients/caregivers, including having their phone contacts
• 24 h access to the team through phone calls and WhatsApp
• Collaboration with support groups that provide basic materials for care such as fluids, analgesics, antimalarial, and others
• Special subsidy on some services such as laboratory tests
• Organizing social events such as end-of-year parties

Table 4 highlights the challenges and burdens of SCD in Nigeria from the perspectives of policymakers. It also enumerates government policies and programmes to address challenges, such as the creation of a National Desk for SCD at the Nigeria Federal Ministry of Health to coordinate all SCD-related activities, the development of National Guidelines for the Management and Control of SCD, the development of Protocol for Universal Newborn Screening for SCD and Expansion of the National Immunization Schedule to include pneumococcal and influenza vaccination for children with SCD.

**TABLE 4:** Feedback from Policy makers (Hospital management staff, Government representative from the Federal Ministry of Health).

Challenges/Burden of SCD in Nigeria	Government policies and programmes to address the challenges
• The 2018 National Demographic Health Survey (NDHS) report for the country put the prevalence of SCD to be highest in the South West (2%), lowest in the South (0.3%) and 21% HbAS and 5% HbAC for southwest and overall prevalence of 1% among the children below 5 years	• Establishment of six zonal Centres of Excellence for SCD which are equipped with HPLC for early detection and comprehensive care of diagnosed babies
	• Creation of a National Desk for SCD at the FMoH
	• Review of the existing National Guideline for the Management and Control of SCD
• SCD is among the top 10 non-communicable diseases (NCDs) causing significant disability, morbidity and mortality impacting negatively on the attainment of Sustainable Development Goals (SDGs) 1, 3, 4 and 10	• Protocol for the Universal Newborn Screening for SCD
	• Expansion of the national immunization schedule to include pneumococcal and influenza vaccination for children with SCD
• Poor integration of SCD prevention and control services with other health and non-health services especially maternal and child health services	• Integration of SCD control and prevention interventions into other relevant intervention activities
• Cultural beliefs and ignorance (myths) about SCD across the country	• Launching of the National Multisectoral Action Plan for SCD and other Prioritized NCDs
• Too many uncoordinated activities of NGOs in SCD community in Nigeria	• Streamlining and coordinating activities of NGO/CSOs in the SCD community
• Non-prioritization of SCD due to poor understanding of the contribution and linkage of the disease to poverty and mortality indices in Nigeria by the major development partners	• High level advocacy resulting in legislative frameworks that ensure SCD is accorded priority attention required considering the high burden of the disease
• Inability of government to mobilize the much-needed resources for SCD interventions	• Multilateral collaboration and partnerships with international organization such as WHO and local industries and NGOs
	• Scaling up high level advocacy and dialogue for SCD

Table 5 shows the number (133), composition and educational level of stakeholders who responded to the survey on knowledge of SCD. Healthcare providers were the most represented, 74 (55.6%), followed by the patients, 32 (24.1%). Other categories of respondents were caregivers 18 (13.5), policy makers 3 (2.3%) and others 6 (4.5%). The majority, 119 (89.4%) had tertiary education.

**TABLE 5:** Socio-demographic data of the participants.

Stakeholders category	Frequency	%
Patients	32	24.1
Caregivers (Parents/Siblings/Cousins)	18	13.5
Medical workers	74	55.6
Policy makers	3	2.3
Other stakeholders (NGO, Media)	6	4.5
Total	133	100
**Educational level**		
Postgraduate degree	74	55.6
Undergraduate degree	45	33.8
Secondary School	12	9.0
Primary School	1	0.8
None	1	0.8
Total	133	100

The majority of the participants had good knowledge of what causes SCD (96.2%) and best place to get help during an SCD crisis (98.5%). In addition, 125 (94.0%) knew that SCD can be prevented and 131 (98.5%) also knew that SCD even if it occurs, can be managed successfully without curing it. However, knowledge on the specific preventive measures of SCD and its crisis was not optimal. For instance, only 17 (12.8%) knew that intake of lots of water is cardinal to crisis prevention, ditto for routine clinic attendance (34.6%) and routine drug use (47.4%). Even, 4 (3.0%) of the stakeholders indicated that patients could stop all medications and clinic visits if they could believe in themselves that they had been healed of SCD.

Knowledge of other measures to reduce the prevalence of SCD was also poor. Less than 10% knew about gene editing and selection, prenatal diagnosis and genetic counseling, less than 20% knew about the role of public awareness, sensitization and education. On the other hand, about half of the participants, 67 (50.4%) indicated that the disease could be prevented when two people who are sickle gene carriers (haemoglobin genotypes AS/AS or AS/AC) refrained from having children.

In terms of roles of community engagement and education, only about one-quarter of the study participants, 34 (25.6%) knew about their positive roles in reducing prevalence of SCD and alleviating SCD crises. One hundred and fourteen (85.7%) however, indicated that community engagement and education may reduce stigmatization of people with SCD.

## Discussion

This study examined the perspectives of critical stakeholders, including patients; parents/caregivers; healthcare providers such as doctors, nurses, pharmacists, medical laboratory scientists, health records officers; and policy makers, on the challenges that patients face in accessing care and best practices that will improve healthcare services. The deliberations of these stakeholders focused on how to increase the number of patients who are enrolled and followed up actively in the SCD clinics at the SPARC-NEt Sites. The study identified factors that might explain the observed inability to implement evidence-based healthcare system policy changes. In broad term, the study showed broad consensus among the groups on strategies for improving healthcare performance.

Generally, patients, parents/caregivers and sickle cell-related non-governmental organizations (NGO) identified delay in accessing care as a major obstacle (Table 2). The delay is both institutional and attitudinal. Institutional delay included the few number of specialist SCD centres coupled with exhaustive and tiring bureaucracies in accessing healthcare. Attitudinal issues identified, included unfriendliness of healthcare providers and providers who were too much in a hurry, to the extent that they could not offer quality care. They also identified that SCD management tended to focus on physical health alone at the expense of social, mental and spiritual health of affected individuals. All the concerned stakeholders agreed that more dedicated specialist SCD centres must be established in the country with more staff and capacity building of the healthcare providers should be prioritized to limit some of these challenges. Similar observations were made by stakeholders (social workers, health workers, child and youth workers, cleaners and policy officers) on the barriers to accessing healthcare services in three rural settings in South Africa (Chinyakata et al., 2021). They reported limited or lack of healthcare facilities and personnel, shortages of medicine, distrust of the healthcare providers, late opening hours of healthcare facilities and financial constraints as major barriers to quality healthcare access in the localities (Chinyakata et al., 2021).

Our study also found that patients and caregivers observed that apart from inefficient emergency SCD care such as blood transfusion services, cost implications of drugs for outpatient care are prohibitive and medications were always out of reach of patients. They reported that although, the National Health Insurance Scheme (NHIS) operates in many secondary and tertiary healthcare centres in the country, the scheme is unfriendly. In a study on the association between level of poverty and SCD severity in a low-resource setting, it was reported that rather than relying solely on medical procedures such as determination of genetic and laboratory markers of disease severity and use of sophisticated equipment, policies that will improve the socioeconomic status of people living in developing countries may offer far reaching influence on SCD complications (Bello-Manga et al., 2020).

Previous studies have shown that more patients with SCD than counterparts non-SCD patients exhibited poor health seeking behaviour in Nigeria. Many patients with SCD sought care from non-hospital and unorthodox places including patent medicine vendors, herbal or traditional medicine shops and faith-based centers including prayer homes and other mind-body therapies in Nigeria (Gideon and Chioma, 2015; Busari and Mufutau, 2017; Olatunya et al., 2021). All participating healthcare providers (doctors, pharmacists, nurses, medical laboratory scientists and health record officers) agreed that routinely evaluated and age-appropriate health education is a very important component of SCD care (Table 3). They believed that it promotes wellbeing through behavioral changes. Hence, efforts should be geared towards improving SCD health literacy levels among patients, for better health-seeking behaviour and better self-care.

Healthcare providers also recommended sickle cell daycare service model as a means of improving access to SCD care. They opined that this option of care should be strategically located within the hospital to provide short-term emergency department (ED) care which cuts down length of hospital stay and cost of care. The model has been practiced with success in developed countries (Ware et al., 1999; Wright et al., 2004; Adewoye et al., 2007; Cline et al., 2018; Olatunya et al., 2021). However, in lower- and middle-income countries, there has been a dearth of knowledge on the use of daycare services such as short-term ED observation care for managing patients with SCD and vaso-occlusive crisis. Adegoke et al. (Adegoke et al., 2020) reported that 96.7% of all hospital visits in a tertiary health facility in Nigeria were managed by short-term ED observation care. The median length of hospital stay was 10.5 h and in 50.3% of encounters, patients were successfully managed without requiring further care. In 17.4% of such admissions, patients had their ED observation care terminated and converted to full admission, and the overall return rate for acute care within 1 week for either persistence of symptoms or any other complaint was 31.7%. The authors concluded that dedicated and protocol-driven short-term ED observation care has the potential to provide effective and timely management of acute pain in children with SCD.

Our stakeholder engagement meeting revealed promising feedback from policymakers, that is, hospital management staff and the government representative from the country’s Federal Ministry of Health (Table 4). All the participating policymakers recognized SCD as one of the top ten non-communicable diseases causing significant disability, morbidity and mortality and impacting negatively on the attainment of Sustainable Development Goals 1, 3, 4, and 10 in Nigeria. They also agreed that poor integration of SCD prevention and control services with other health and non-health services especially maternal and child health services, as well as the inability of the government to mobilize the much-needed resources for SCD interventions and uncoordinated activities of NGOs and researchers on SCD in Nigeria, were issues that needed to be dealt with for much-needed progress to be made in improving SCD care (Nnodu Obiageli et al., 2020; Nnodu et al., 2021).

The policymakers highlighted some of the current efforts of the government at alleviating challenges facing SCD care in the country (Table 4). These included the creation of a National Desk for SCD at the FMoH, development of the National Guideline for the Management and Control of SCD, development of Protocol for the Universal Newborn Screening. They also listed the establishment of six zonal Centres of Excellence for SCD which were equipped with High Performance Liquid Chromatography (HPLC) machines for early detection and comprehensive care of diagnosed babies. Currently, the national immunization schedule has been expanded to include pneumococcal and influenza vaccination for children with SCD; and activities of NGO/CSOs in the SCD space have undergone intense streamlining and coordination by the Ministry of Health such that broad-based NGOs like the Sickle Cell Support Society of Nigeria (SCSSN) is a major stakeholder and technical partner of the Federal in matters related to SCD in the country. Government is also encouraging multilateral collaboration and partnerships with international organization such as World Health Organization, local industries and NGOs. Lastly, high level advocacies are ongoing which will ultimately result in legislative frameworks that will ensure SCD is accorded priority attention in the country.

Knowledge of the participants on different measures to the reduce prevalence of SCD was found in this study to be poor (Table 6). A small proportion knew about gene editing and selection, prenatal diagnosis and genetic counselling, roles of public awareness, sensitization and education. Worse still was the fact that supposed stakeholders did not know much on the roles of community engagement and education, in reducing the prevalence of SCD and alleviating SCD crises. These findings underscore the need for regular and robust stakeholder engagement meetings to improve knowledge and perspectives of stakeholders.

**TABLE 6:** Knowledge of the stakeholders about SCD.

Knowledge	Frequency	%
**What causes SCD**		
• Genetic inheritance	128	96.2
• Inherited sin, mating at the wrong time	3	2.3
• Punishment from God	2	1.8
**Best place to get help during crisis**		
• Hospital or Clinic	131	98.5
• Church for prayers, herbalist, chemist or pharmacies	2	1.5
**Are there things that you can do to prevent crises and illness?**		
• Yes	128	96.2
• No	5	3.8
**Things that can be done to prevent crises and illness**		
• Drinking a lot of water	17	12.8
• Going for routine clinic regularly	46	34.6
• Taking your routine drugs regularly	63	47.4
• Believing that you have been healed and stopping all medications and clinic visits	4	3.0
**Can sickle cell disease be cured?**		
• Yes	73	54.9
• No	57	42.9
**Can sickle cell disease be managed successfully without curing it?**		
• Yes	131	98.5
• No	2	1.5
**Can sickle cell disease be prevented?**		
• Yes	125	94.0
• No	3	2.3
**Sickle cell disease can be prevented by**		
• Community engagement and education about it	34	25.6
• Legislation that prevents carriers from marrying each other	29	21.8
• Two people who are carriers (AS/AS or AS/AC) refraining from having children	67	50.4
**Other methods of preventing SCD**		
• Gene editing and selection	3	2.3
• Legislation to prevent marriage between carriers	10	7.5
• Prenatal diagnosis	4	3.0
• Genetic counselling	11	8.3
• Through public awareness, sensitization and education	23	17.3
**What is the best strategy to remove stigmatization of people with sickle cell disease?**		
• By community engagement and education about it	114	85.7
• By legislation	11	8.3
**What are patient support groups for SCD?**		
• Government groups that support patients financially	21	15.8
• Doctors reaching out to patients	6	4.5
• Patients and other stakeholders coming together to support patients	98	73.7
• NGOs	3	2.3
**How do patients with SCD pay their bill? (tick all that apply)**		
• Help from support groups	6	4.5
• Help from relatives	18	13.5
• Health insurance	7	5.3
• Out of pocket	96	72.2
• NGOs	6	4.5

## Conclusion

The major challenges to access to healthcare services identified by stakeholders were difficulty and delays in accessing specialist SCD care, poor communication, uncoordinated and high cost of healthcare services. Other challenges include high burden of the disease, poor integration of SCD prevention and control services within the health and non-health sectors, non-prioritization of SCD, poor advocacy, too many uncoordinated SCD-related NGOs and poor knowledge of the role of stakeholders’ engagement. Solutions proffered by stakeholders to improve healthcare services for patients with SCD include the provision of more specialist centers and capacity building of healthcare personnel, improvement of emergency and blood transfusion services and increased health insurance coverage. Others include health education and genetic counseling, adoption and implementation of the SCD Day Care Service Model in all centers, collaborations with sickle cell support groups, providing 24-hour access to patients through cell phones and social media Apps platforms and implementing the outlined government policies and programmes.

## Data Availability

The original contributions presented in the study are included in the article/supplementary material, further inquiries can be directed to the corresponding author.
